# Cardiac surgery in an infant hemophilia B carrier with moderate hemophilia: a case report

**DOI:** 10.3389/fcvm.2026.1690797

**Published:** 2026-03-18

**Authors:** Julia Hölz, Chiara Nobile, Johannes Holzapfel, Victoria Lieftüchter, Nicole De Winkel, Martin Olivieri

**Affiliations:** 1 Pediatric Thrombosis and Hemostasis Unit, Dr. von Hauner Childreńs Hospital, LMU Munich, Munich, Germany; 2 Department of Pediatric Cardiology and Congenital Heart Disease, German Heart Centre Munich, Technische Universität Munich, Munich, Germany

**Keywords:** cardiac surgery, hemophilia B, infant, perioperative management, recombinant factor IX, Trisomy 21

## Abstract

**Introduction:**

This is the first reported case of a female infant with moderate hemophilia B undergoing complex cardiac surgery, highlighting challenges in managing bleeding disorders in this rare clinical setting.

**Patient concerns and clinical findings:**

A 4-month-old girl with Trisomy 21 was found to have moderate hemophilia B and an atrioventricular septal defect requiring surgery.

**Diagnosis, interventions, and outcomes:**

Genetic testing confirmed hemophilia B. Surgery was performed successfully using extended half-life recombinant factor IX (rFIX-FP) to maintain stable coagulation. The patient experienced no excessive bleeding and recovered well.

**Conclusion:**

This case demonstrates that extended half-life FIX products enable safe and effective management of moderate hemophilia B during complex cardiac surgery in infants. Individualized perioperative planning and interdisciplinary collaboration are essential for optimal outcomes.

## Introduction

Hemophilia B is a rare recessive, X-linked bleeding disorder caused by deficiency or absence of coagulation factor IX (1), accounting for 10%–20% of all hemophilia cases (2). The prevalence is approximately 3.8 cases per 100,000 males (3). Primarily affecting males, low factor IX levels in females can be due to six causes: (1) homozygosity, (2) compound heterozygosity, (3) hemizygosity, (4) heterozygosity, (5) other genetic causes, and (6) non-genetic causes (4). Alternatively, the World Federation of Hemophilia further classifies carriers as asymptomatic or symptomatic with mild, moderate, or severe hemophilia (5). Severely and moderately affected female carriers are very rare. To date, only approximately 250 women and girls with a severe or moderate phenotype have been reported in the literature (4, 6).The standard therapy regimen consists of factor replacement with plasma-derived or recombinant FIX concentrates, either in a prophylactic scheme or as an on-demand treatment in case of bleeding, depending on hemophilia severity (7). The coexistence of congenital heart disease and hemophilia is extremely rare. The incidence of congenital heart disease does not differ among the hemophilic population compared to the general population (8). While several cases of cardiac surgery in hemophilia A infants have been reported (9), there is only one documented case of cardiac surgery in a male infant with hemophilia B (10). Despite well-established daily treatment regimens for hemophilia B, little is known about the management of infants undergoing cardiac surgery and the subsequent perioperative care.

## Case report

**Figure 1:**
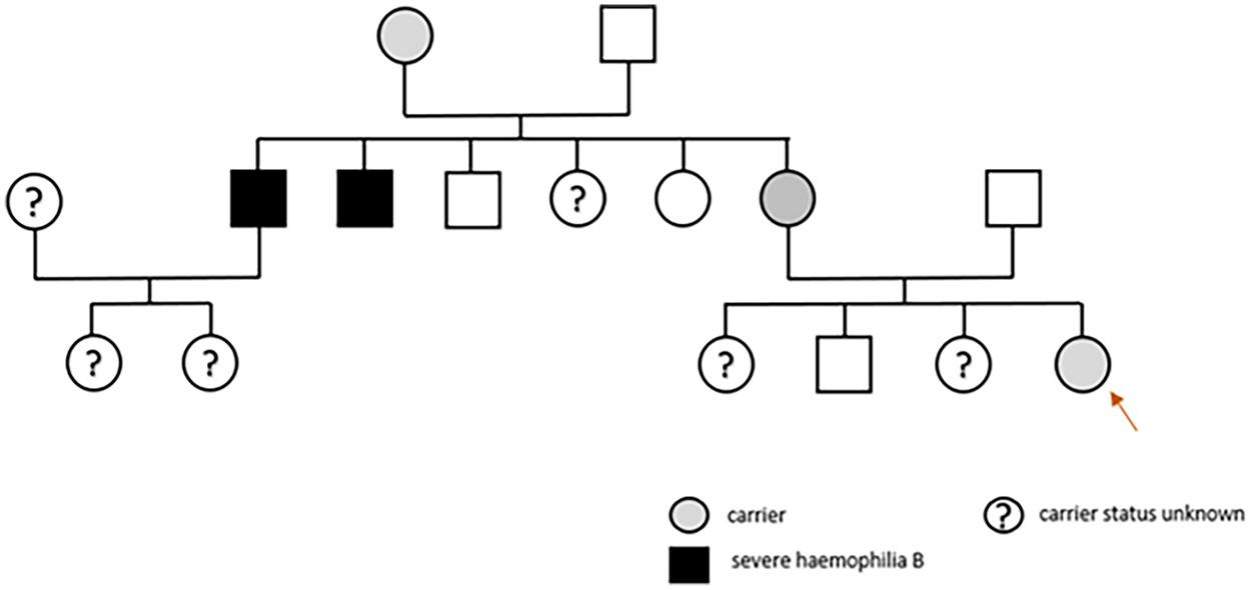
Family tree of the patient (marked with red arrow); gray filled circle: conductor with genetic variants in exon 1 and exon 8; black square: severe hemophilia B (not genetically tested).

We report the rare case of a female infant (4 months at the time of surgery) with Trisomy 21 and moderate hemophilia B, who also presented with an atrioventricular defect (Rastelli type A). There is a strong association between Trisomy 21 and congenital atrioventricular septum defect (CAVSD), with 40% of all fetuses diagnosed with AVSD in utero also having Trisomy 21 (11). Despite a family history of severe hemophilia B in two maternal uncles, the mother’s hemophilia B carrier status was not tested. In the preoperative workup, a prolonged aPTT of 63 seconds and FIX levels ranging between 3% and 6% (one-stage assay) were measured in the infant girl. No bleeding symptoms were reported by the family. The patient’s International Society on Thrombosis and Hemostasis Bleeding Assessment Tool (ISTH BAT) score was zero. Genetic testing revealed two mutations (c.19A>T, c.1178_1180delACA), in exon 1 and exon 8 of the FIX gene, confirming a diagnosis of moderate hemophilia B. The mother carried the same mutations, with had significantly higher FIX levels (33%–41%). The father had no relevant mutation in the FIX gene (Figure 1). The differing FIX levels between mother and daughter could be due to preferential or skewed X chromosome inactivation, which is reported in the literature on rare occasions (12). To analyze the inactivation pattern of the X chromosomes, CAG tandem repeats within the androgen receptor were investigated. Interestingly, the X chromosome inactivation analysis showed random inactivation with a ratio of 51%–49%. In addition, the immature hemostatic system during the first year of life, characterized by markedly lower coagulation factor levels compared with later life, could represent an alternative explanation. However, at the age of 2 years, her FIX levels remained at 5% as measured by chromogenic assay.

The planned cardiac surgery involved closure of the ventricular septal defect (VSD) with a Goretex patch, closure of the atrial septal defect (ASD) with a pericardial patch, cleft suture of the left atrioventricular valve, closure of the patent foramen ovale, and clip closure of the patent ductus arteriosus. Risk factors for bleeding included cardiopulmonary bypass, systemic therapeutic anticoagulation with unfractioned heparin, and protamine reversal. To minimize both bleeding and thrombosis risk, a prolonged half-life rFIX product (Albutrepenonacog alfa, rFIX-FP, Idelvion®), a recombinant fusion protein of recombinant factor IX and albumin, was used to maintain stable FIX concentrations with fewer infusions. The aim was to ensure preoperative coagulation levels comparable to those of a healthy individual, thereby allowing for standard cardiopulmonary bypass, systemic heparinization, and protamine reversal. The treatment plan was discussed with the anesthesiology, cardiac surgery, and pediatric cardiology departments, as well as with the patient’s parents.

Preoperatively, 500 IU rFIX-FP (100 IU/kg body weight) was administered. The operation lasted 224 min, with an extracorporeal bypass time of 115 min. During the procedure, the patient received 660 mL RBC, 185 mL FFP, and 1 g fibrinogen concentrate. Eight hours postoperatively, her FIX level was 115% (one-stage assay), and additional 250 IU FIX (50 IU/kg body weight) was administered. For therapeutic anticoagulation, 5000 IE/m^2^/day of heparin was used.

**Figure 2:**
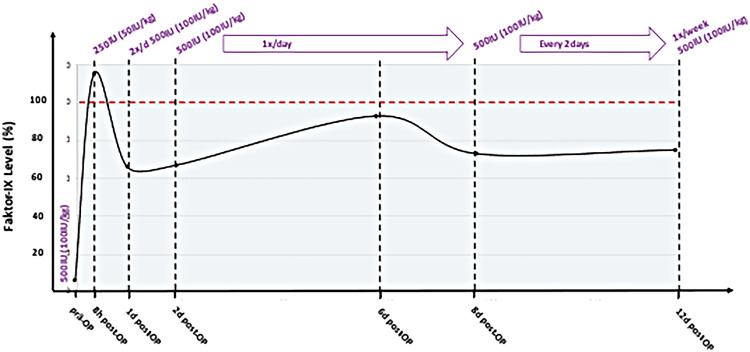
Preoperative and postoperative monitored FIX levels and dosage of rFIX-FP.

Periprocedural and postoperative FIX activity levels in relation to rFIXFc administration are illustrated in Figure 2.

The intraoperative bleeding in this patient was comparable to that of a non-hemophilic patient undergoing this procedure. Patient-specific complications included AV block with intermittent pacemaker indication and an unstable thorax with mediastinitis. The patient was discharged within 3 weeks following surgery. We continued the once-weekly prophylaxis with rFIX-FP. To date, no FIX inhibitor has been detected.

## Discussion

This is the first published report of a female patient with moderate hemophilia B undergoing cardiac surgery. To date, only one case of an infant with hemophilia B undergoing cardiac surgery has been reported in the literature (10). That surgery was carried out in a resource-limited setting (Sudan), limiting comparability with results in developed countries. The present case study shows that extended half-life FIX products are safe for use in complex cardiac surgery with cardiopulmonary bypass, as well as in postoperative care. Regular factor IX measurements and dose adjustment are important components of the treatment strategy. Nevertheless, continuous infusion of factor IX is also a viable approach. We chose single doses over continuous factor IX infusion because of our team’s better experience with this method. We opted for an extended half-life FIX product rather than a standard half-life FIX product, as the latter requires more frequent administration and may result in fluctuating factor IX levels. This case shows that good preoperative coagulation leads to a comparable risk of bleeding despite hemophilia. It therefore can contribute to the standardization of therapy for infant hemophilia patients undergoing cardiac surgery. In addition, this case is an example of the importance genetic testing in families with known hemophilia, especially as the patient's mother was not aware of her carrier status and her father had no bleeding history. In hemophilia carriers, molecular testing remains the only way to provide an accurate diagnosis, overcoming the variability and inconsistent data obtained from other methods based on the quantification of coagulation factors (6). The process of X inactivation in humans is still not well understood. Even if we could not prove skewed X inactivation, it is known that partial X inactivation occurs in various cell types and organs (13). Furthermore, it has been shown that the degree of X inactivation is correlated with the level of factor IX in fraternal female twins (14). Therefore, a partial inactivation of the X chromosome in hepatocytes—which are the exclusive factor-IX-producing cells—remains the most plausible explanation. For ethical reasons, confirmation through liver biopsy is not feasible.

## Conclusion

This case represents the first reported instance of successful cardiac surgery in a female infant with moderate hemophilia B, Trisomy 21, and AVSD. It highlights the feasibility and safety of using extended half-life rFIX products in complex cardiac procedures. Individualized perioperative planning, interdisciplinary collaboration, and targeted genetic testing in at-risk families are critical. Furthermore, this case underscores the diagnostic limitations of relying only FIX activity levels in female carriers and emphasizes the importance of molecular testing, particularly in the context of variable X chromosome inactivation.

## Data Availability

The raw data supporting the conclusions of this article will be made available by the authors, without undue reservation.
